# Children's Wards

**Published:** 1908-11-21

**Authors:** 


### The Hospital

A Journal of

The Medical Sciences and Hospital Administration.

New Series. No. 89, Vol. IV. [No. 1161, Vol. XLV.] SATURDAY, NOVEMBER 21, 1908.

### CHILDREN'S WARDS.

The specialisation of hospitals is a subject on which much ink has been expended and a great deal of interesting discussion has taken place. For all that, it is highly improbable that the final word has yet been said. Certain principles have been agreed to, but that is about all. We have consented, for example, to look with disfavour on mixed hospitals, and the infectious diseases ward in a general hospital is an anachronism that no amount of argument can condone. Still we have not progressed so far that we regard those hospitals as absolutely antiquated that have no special wards for syphilitic patients or for the tuberculous. Both these classes are admitted into the general wards in nearly all our large hospitals, although the modern tendency is distinctly towards isolation and separation in both cases. Equally uncertain is our attitude with regard to children's wards. At present there are not a few institutions, among them some of our large metropolitan hospitals, in which no special wards for juvenile patients are to be found. Child patients are accommodated in cots placed in the general wards, and it is no uncommon sight to see a baby with hare-lip at one end of the room and an old patient with a senile complaint at the other. The only distinction that appears to be made is in the allotment of cots in the male or female wards, and there the bare age of the child is considered. Male children over seven years of age are relegated to the male side, and the result of this subtle distinction is that the male wards are deprived of whatever advantages the presence of infants may confer upon them, while they are spared the nursery horrors which are the lot of the patients on the female side. This distribution of child patients is defended by many on the old plea that the women like the presence of children, and that at the worst the baby in the cot may be a distraction to its fellow-sufferers. The added argument that number So-and-so may in the nurse's absence keep an eye on the cleft palate at her side, or even nurse it in bed, is one that we do not imagine any sensible person will favour. Hospital patients should not be made to act as children's nurses, and it is unpardonable to permit children in a ward where nursing economy is driven to such excess that a special nurse cannot be told off to watch a child whose condition demands constant attendance and care. The objections to mixed juvenile and adult wards are many; the pleas in their favour are weak, and for the most part sentimental. That children may serve to distract the adult patients' attention we do not presume to deny, and the presence of children in the day room or on the balcony can hardly be anything except beneficial to both classes, adults and juveniles. But in the wards themselves it is different. There the sick adult is more likely to curse the fate that assigned him the post of bed neighbour to a squalling infant than to bless the presence of the cherub that comes up to offer him a grubby and forbidden dainty. Children's minds are impressionable, and to allow them to be moulded and indelibly impressed by the things they may see and hear in a general ward is, to say the least, injudicious.

For these reasons the children's ward in a general hospital is a subject which demands the careful and solicitous consideration of the committee. No institution is too old or too poor to make some provision, small and mean though it may be, for the benefit of its juvenile patients. No institution, again, deserves to have juvenile patients until and unless it can make such provision. Many hospitals have struggled on for years under the old conditions, and the necessity for a special children's ward would be heatedly debated by their staffs, both medical and nursing. Old prejudices die hard, and one of the most rooted of opinions held by some hospital officials is that the mixing of young and old patients in a general ward conduces to the benefit, moral and otherwise, of such a ward. That is an opinion which no one who regards the matter frankly and with an open mind can honestly share; and the best argument against it is to spend a week, as a patient, in a ward where children are admitted. A sick adult does not look with favour on the nursery. In ordinary practice we exclude the children from the sick-room. In hospital practice we are asked to reverse the process and to agree that an adult patient has blunter feelings and deafer ears than he has outside the institution.

The provision of children's wards in a general hospital is, then, a matter which is worthy of special attention, and to this may be added that it is equally necessary to see that such wards, when provided, are suitable for the accommodation of the class of patients they are destined to receive. A popular notion of a children's ward appears to be a badly-ventilated room which has its walls covered with posters illustrative of nursery rhymes. Fresh air, and much of it, are more necessary in such wards than the most elaborate Kate Greenaway designs; overcrowding and cramped bed space will exact a death toll far heavier here than in the adult wards. The main principles that should be regarded in the construction and fitting up of children's wards are those that have been admirably followed in many of our children's hospitals. These are not models to be slavishly followed in designing a children's ward for a general hospital, but they are examples which should at least be considered. In many cases, especially where our older hospitals are concerned, it would need a great deal of skill and not a little thought to design a separate children's ward that should harmonise with the rest of the structure and adequately serve its purpose. Yet we venture to think such wards should be designed, for their usefulness is obvious, and they will do away with the many objections which can be raised against the present system of mixing young and old sufferers in a common ward without regard to age or anything else except the fact that all are patients.

